# Obstruction of Bowel, Probably Intussusception, Relieved by Aspiration

**Published:** 1880-04

**Authors:** Henry F. Godfrey

**Affiliations:** Benton, Wis.


ARTICLE VIII.

*Obstruction of Bowel, probably Intussusception, relieved by Aspiration.*

Oct. 21st, 1879, was called in consultation with my friend Dr. Fowler, of Scales Mound, Ill., to visit William Bird, aged 37, an English miner. A week previous to my visit he spent the evening at a friend's house, returned home in apparently good health, and went to bed. Was taken suddenly in the night with severe pain in the bowels and vomiting. Supposing the case to be cholera morbus, or some similar disease, he took some of the usual domestic remedies, but failing to obtain relief, called Dr. Fowler, his family physician, on the following evening.

The nature of the case was at once recognized by this gentleman, who prescribed appropriate treatment.

He was shortly afterwards seen by Dr. August Weirich, of Galena, who confirmed Dr. Fowler's diagnosis of obstruction from a band or intussusception. He gradually got worse, and when I first saw him was laid in bed on his back, partly dressed, knees drawn up, the pulse was quick and small, the countenance pinched and anxious. There was constant vomiting of a greenish yellow liquid, with a horribly offensive fæcal odor, which penetrated the whole house. The abdomen was swollen and quite tender. There was distinct fluctuation and dullness on percussion over the whole course of the colon. The umbilical region gave a slightly tympanitic resonance. There was very frequent tenesmus which caused the patient to jump up constantly and strain on the vessel, the only result being a little bloody mucus.

After careful examination for hernia, which I did not find, I diagnosticated an obstruction at or near the sigmoid flexure, and proposed aspiration of the colon, an expedient which was readily agreed to by the patient and his friends.

I used the largest needle of Codman & Shurtleff's aspirator, puncturing first in the left and afterwards in the right iliac region, drawing off a pint of liquid similar in character to that vomited, but more concentrated, the vomited matter being diluted with water taken to quench the constant tormenting thirst. I directed a seidlitz powder to be given by enema, injecting first the alkaline powder and follow it immediately by the acid. I hoped by this means (the pressure of liquid being taken off from above) to dilate and unfold the bowel, and also to obtain the sedative effect of the carbonic acid on the bowel. The material necessary was not at hand, and as I learned afterwards, the enema was not efficiently given, no plug or pad was used and the nascent gas escaped at once per anum.

Immediately after the aspiration, at 3 p. m., he declared himself somewhat relieved. The vomiting continued at longer intervals till 9 o'clock, when, under the influence of a hypodermic injection of morphia administered by Dr. Fowler, he fell asleep, and had a comfortable night.

I saw him again at noon on the 22d. He had not vomited since nine the previous night, his abdomen was natural in appearance, all swelling and tenderness had disappeared, no tenesmus, and he had more appetite than it was thought prudent to gratify. The effect of the morphia was continued, by small doses, by the mouth. His bowels moved on the third day, and he was able to be out in less than a week.

I may add, that this result was a pleasant surprise, both to myself and Dr. Fowler, and I desire to put it on record as a simple and innocuous proceeding which may be tried before proceeding to graver operations demanded by this formidable accident. Very respectfully, HENRY F. GODFREY, M.D.

BENTON, WIS., Feb. 13, 1880.

